# Associations between sleep duration, sleep disturbance and cardiovascular disease biomarkers among adults in the United States

**DOI:** 10.1186/s12889-024-18381-5

**Published:** 2024-04-02

**Authors:** Prince Nii Ossah Addo, Paddington T. Mundagowa, Longgang Zhao, Mufaro Kanyangarara, Monique J. Brown, Jihong Liu

**Affiliations:** 1 Department of Epidemiology and Biostatistics, Arnold School of Public Health, University of South Carolina, 915 Greene Street, 29208 Columbia, South Carolina USA; 2 Arnold School of Public Health, South Carolina Smart State Center for Healthcare Quality, University of South Carolina, Columbia, South Carolina USA; 3 Rural and Minority Health Research Center, Arnold School of Public Health, University of South Carolina, Columbia, South Carolina USA; 4 Office for the Study on Aging, Arnold School of Public Health, University of South Carolina, Columbia, South Carolina USA

**Keywords:** Sleep duration, Sleep disturbance, Biomarker, Cardiovascular diseases, NHANES

## Abstract

**Background:**

Sleep problems are associated with abnormal cardiovascular biomarkers and an increased risk of cardiovascular diseases (CVDs). However, studies investigating associations between sleep problems and CVD biomarkers have reported conflicting findings. This study examined the associations between sleep problems and CVD biomarkers in the United States.

**Methods:**

Data were from the National Health and Nutrition Examination Survey (NHANES) (2007–2018) and analyses were restricted to adults ≥ 20 years (*n* = 23,749). CVD biomarkers [C-reactive Protein (CRP), low-density lipoproteins, high-density lipoproteins (HDL), triglycerides, insulin, glycosylated hemoglobin (HbA1c), and fasting blood glucose] were categorized as abnormal or normal using standardized cut-off points. Sleep problems were assessed by sleep duration (short [≤ 6 h], long [≥ 9 h], and recommended [> 6 to < 9 h) and self-reported sleep disturbance (yes, no). Multivariable logistic regression models explored the associations between sleep duration, sleep disturbance, and CVD biomarkers adjusting for sociodemographic characteristics and lifestyle behaviors.

**Results:**

The mean sleep duration was 7.1 ± 1.5 h and 25.1% of participants reported sleep disturbances. Compared to participants with the recommended sleep duration, those with short sleep duration had higher odds of abnormal levels of HDL (adjusted odds ratio [aOR] = 1.20, 95% confidence interval [CI] = 1.05–1.39), CRP (aOR = 3.08, 95% CI = 1.18–8.05), HbA1c (aOR = 1.25, 95% CI = 1.05–1.49), and insulin (aOR = 1.24, 95% CI = 1.03–1.51). Long sleep duration was associated with increased odds of abnormal CRP (aOR = 6.12, 95% CI = 2.19–17.15), HbA1c (aOR = 1.54, 95% CI = 1.09–2.17), and blood glucose levels (aOR = 1.45, 95% CI = 1.07–1.95). Sleep disturbance predicted abnormal triglyceride (aOR = 1.18, 95% CI = 1.01–1.37) and blood glucose levels (aOR = 1.24, 95% CI = 1.04–1.49).

**Conclusion:**

Short and long sleep durations were positively associated with abnormal CRP, HDL, HbA1c, blood glucose, and insulin levels, while sleep disturbance was associated with abnormal triglyceride and blood glucose levels. Since sleep is a modifiable factor, adopting healthy sleeping habits may create a balanced metabolism and reduce the risk of developing a CVD. Our study may provide insights into the relationship between sleep duration, sleep disturbance, and CVD risk.

**Supplementary Information:**

The online version contains supplementary material available at 10.1186/s12889-024-18381-5.

## Introduction

Cardiovascular diseases (CVDs) refer to a group of diseases that affect the heart and blood vessels and are the leading cause of mortality and morbidity globally. In 2019, 38% of premature deaths in people under 70 years were caused by CVDs [1]. Between 2017 and 2018, 12% of health expenditures in the United States (U.S.) were used to treat and control CVDs [2]. It is projected that by 2035, 45.1% of the U.S. adult population will have at least one CVD, and the total medical costs of CVDs will reach $1.1 trillion [3]. CVDs are an important public health challenge in the U.S. However, since some predictors of CVDs, such as sleep problems, are lifestyle-related (modifiable), the risk of developing a CVD could be reduced through lifestyle changes.

Consistently, studies have evidenced a high prevalence of sleep problems among the U.S. adult population [4, 5]. More than one-quarter of U.S. adults slept less than the recommended hours [5], and 14.5% of U.S. adults had trouble falling asleep in 2020 [4]. Short sleep duration (≤ 6 h), long sleep duration (≥ 9 h), and poor sleep quality have been associated with an increased risk and higher incidence of CVD [6, 7]. Sleep deprivation impairs coronary microcirculation [8], disrupts endothelial functioning and causes early decline of vascular structure and function, which may increase CVD risk [9]. Sleep deprivation also impairs glucose homeostasis and insulin sensitivity, increasing CVD risk [10]. Since sleep problems are modifiable, changes to sleep patterns have the potential to prevent or delay the onset of CVDs and are therefore essential lifestyle factors to consider in the fight against CVDs. Another important factor in fighting CVDs is the early detection of people at a higher risk of developing CVDs. Several biomarkers influence CVD risk and are therefore crucial in the early detection and prevention of CVDs. Clinically relevant biomarkers that have been used as markers of CVD risk include C-reactive Protein (CRP) [6], low-density lipoproteins (LDL) [11], high-density lipoproteins (HDL) [11], total cholesterol (TC) [6], triglycerides (TG) [6], insulin [7], glycosylated hemoglobin (HbA1c) [7], and fasting blood glucose [7]. Therefore, early detection of CVD risk using biomarkers could help reduce the incidence of CVDs and their associated health and economic consequences in the U.S.

A few studies have investigated the association between sleep duration, sleep disturbance, and CVD biomarkers, and these studies have reported conflicting findings. Some of these studies used the NHANES data but focused primarily on CRP. For instance, Grandner et al. (2013) examined the association between sleep duration and CRP among U.S. adults aged 18–80 years using the NHANES data (2007–2008). They reported associations between short and long sleep duration and higher CRP levels [12]. Another study using the NHANES data (2007–2010) to examine the same associations among U.S. adults reported higher CRP levels in men with short sleep duration [13]. Another study associated long sleep duration with higher CRP levels among women [6]. Conversely, a study conducted among adults aged 48 to 92 years in Britain found no significant association between sleep duration and CRP levels [14]. A study conducted among men aged 71–92 years in the United Kingdom reported an association between extreme sleep durations (short and long sleep) and HbA1c, but no association with other CVD biomarkers [7]. Most of the previous studies that have investigated the association between sleep problems and CVD biomarkers have focused on specific age groups (e.g., 40–45 years, 18–25 years) [11], and specific CVD biomarkers (e.g. CRP) [12, 14].

Therefore, we sought to examine how sleep duration and sleep disturbance are associated with CVD biomarkers among a large sample of the U.S. adult population. We hypothesized that long sleep duration, short sleep duration, and self-reported sleep disturbance would be positively associated with abnormal levels of all CVD biomarkers. Knowledge of the association between sleep problems and CVD biomarkers could help design interventions targeting specific sleep challenges of people at high risk of CVDs. Such interventions could lead to positive health outcomes for such individuals.

## Participants and methods

### Data source

This study used data from the National Health and Nutrition Examination Survey (NHANES, 2007–2018). NHANES uses a complex multistage probability sampling design to ensure that the selected sample represents the U.S. population [15]. The survey assesses the health and nutrition status of people of all ages through interviews and medical examinations. The NHANES interview collects data on participants’ general health, socioeconomic and demographic characteristics, while the examination component comprises physical examinations (e.g. anthropometric measurements), clinical measurements, and biospecimen (e.g. blood, urine) collection for laboratory tests [15]. Examinations are performed in customized and equipped mobile exam centers (MEC). Data from NHANES are publicly released in 2-year cycles. We used NHANES data from 2007 because that was when changes were made to the subgroups being over-sampled.

### Study population

**Fig. 1:**
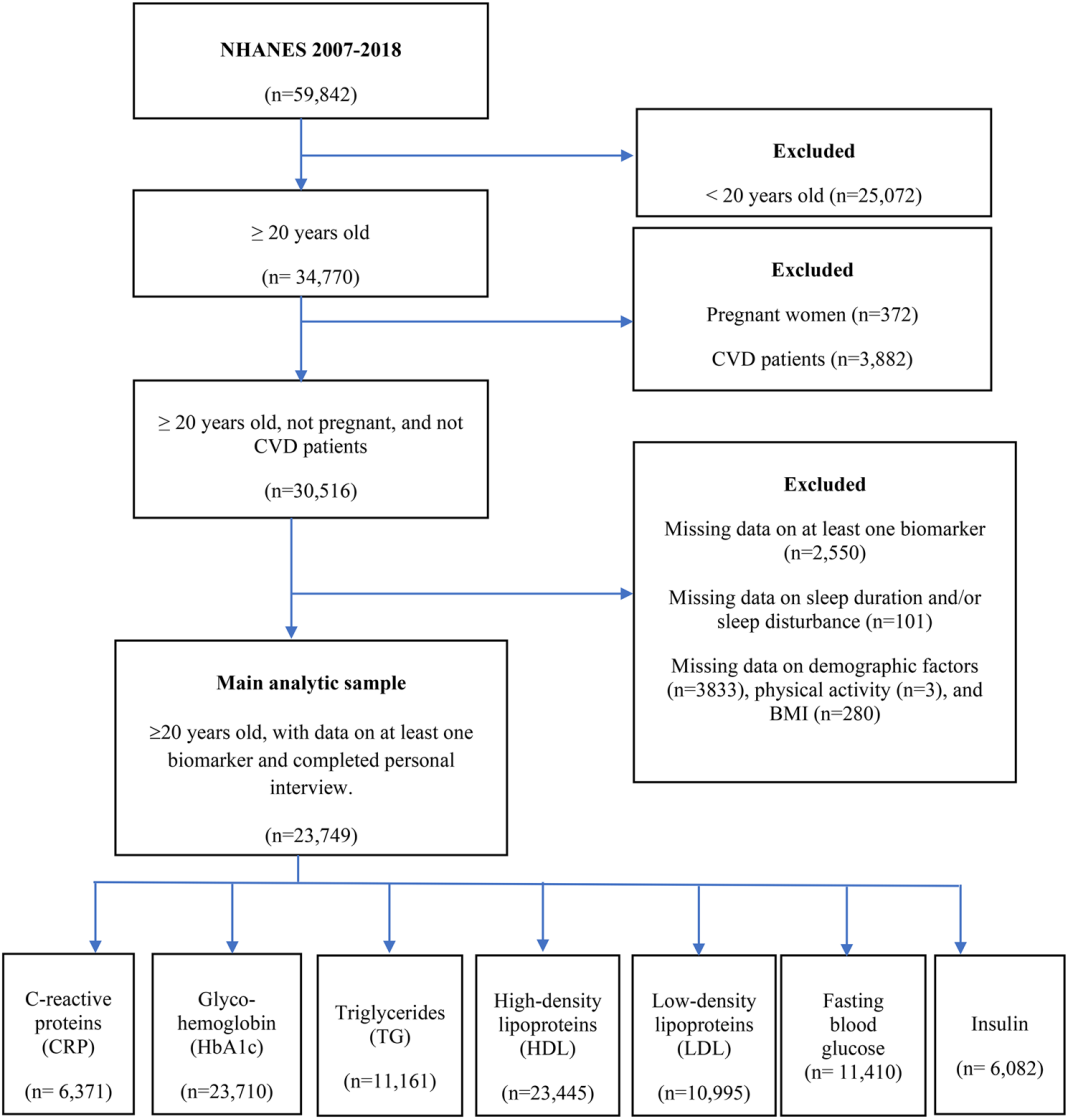
Flow chart showing the final analytic sample used in this study. Abbreviations: CVD- cardiovascular disease; BMI- body mass index

Study participants were adults (≥ 20 years) who participated in the NHANES survey during the six cycles from 2007 to 2018. Participants aged ≥ 20 years were chosen because the NHANES survey consistently collected data for all needed variables for this age group. Participants included in this study were not pregnant, did not report having been told by a doctor that they had a CVD, and had complete data on sleep duration, sleep disturbance, CVD biomarkers, and covariates (described below). We derived a final analytic sample of 23,749 after applying the eligibility criteria above (Fig. 1). Supplementary Table A.1. shows the background characteristics of included and excluded participants.

### CVD biomarkers

The main outcome variables were CVD biomarkers obtained from participants’ fasting blood samples collected at MEC. We used CVD biomarkers associated with sleep measures in the literature, including HDL, LDL, TG, CRP, HbA1c, insulin, and blood glucose. NHANES data collection for CVD biomarkers was consistent throughout the study period except for CRP, which was only available for 4 years (2007–2010), and insulin, which was only available for 6 years (2013–2018). Data on the biomarkers were categorized as abnormal or normal using standardized cut-off points used in previous studies [16, 17]. Participants with LDL ≥130 mg/dL, HDL ≤ 50 mg/dL, CRP ≥ 3 mg/dL, insulin ≥ 10.57 µU/mL, HbA1c ≥ 6.5%, blood glucose ≥ 126 mg/dL [16], TG ≥ 150 mg/dL [17] were categorized as having abnormal levels of the biomarker.

### Sleep duration and sleep disturbance

The main exposure variables were self-reported sleep duration, which was assessed by asking participants the average number of hours they sleep at night on workdays, and self-reported sleep disturbance which was assessed by asking participants if they ever told a health professional that they had trouble sleeping (yes vs. no). Using the same cut-off values for sleep categories as previous studies, sleep duration was categorized as short duration (≤ 6 h), recommended duration (> 6 to < 9 h), and long duration (≥ 9 h) [13, 18]. Participants who responded ‘don’t know’ or ‘refused’ were considered as having missing responses.

### Covariates

We chose covariates from previous studies that have examined the relationship between sleep problems and cardiometabolic biomarkers [12, 14, 18]. The sociodemographic characteristics known to be associated with both CVD biomarkers and sleeping problems were age (20–29, 30–39, 40–49, ≥ 50 years), sex (male, female), race/ethnicity (Hispanic, non-Hispanic White, non-Hispanic Black, Other race), education level (less than 12th grade, high school diploma, some college, college graduate or above) and marital status (never married, married, divorced/widowed/separated, living with a partner) were adjusted for possible confounding influence [18]. NHANES uses the Physical Activity Questionnaire (PAQ) to assess participants’ physical activity through interviews. PAQ was developed from the global physical activity questionnaire and collects information about participants’ daily, sedentary, and leisure-time activities [15]. Physical activity was included as a lifestyle factor that influences sleep quality. Participants were categorized as either physically active or not active according to the Physical Activity Guidelines for Americans (PAG), that is, if they participated in at least 150 min per week of moderate-intensity aerobic physical activity [19]. Body mass index (BMI) was also included as a health status variable influencing sleep duration and sleep disturbance. BMI was classified based on cut-off points recommended by the World Health Organization (WHO) for adult populations [20].

### Statistical analyses

Statistical analyses took into consideration the stratified, clustered, multistage sampling design of the NHANES survey [15]. The 4-year sub-sample MEC weights were used for CRP, 6-year MEC weights for insulin, and 12-year MEC weights were used for the other outcome variables (biomarkers). The Wald Chi-square test for categorical variables was used to test differences in the distribution of covariates by categories of sleep duration and sleep disturbance. Multivariable logistic regression techniques were used to examine the association between categories of sleep duration (short, recommended, and long duration) and each CVD biomarker (CRP, TG, HDL, LDL, blood glucose, insulin, and Hb1Ac) using the recommended duration as the reference. Multivariable logistic regression was also used to examine the association between categories of sleep disturbance and each abnormal CVD biomarker. All models were fit using SAS survey procedures, considering the NHANES complex survey design. We reported both crude and adjusted odds ratios (ORs) with 95% confidence intervals (CI) and conducted sensitivity analysis without assumptions (E-value) [21] to measure the potential effect of unmeasured confounders on the associations. The E-value shows the minimum strength of association an unmeasured confounder would need to have with the exposure and outcome to explain away the observed exposure-outcome association [21]. Possible interactions between each sleep practice and gender, age, or race were investigated. Data were analyzed using SAS software, Version 9.4 of the SAS System for Windows (SAS Institute, Cary, NC). A two-sided P value of less than 0.05 was considered statistically significant. The Institutional Review Board (IRB) considered this study exempt from IRB approval since we used de-identified publicly available datasets.

## Results

### Sociodemographic characteristics of participants

A total of 23,749 participants aged at least 20 years from the NHANES data for the years 2007 to 2018 were used as the main analysis sample (Fig. 1). Table 1 shows the sociodemographic distribution of the study sample. Female participants accounted for slightly above half (52.7%) of the overall sample and the mean age was 48.9 ± 16.9 years. About two-thirds (66.5%) of the participants were non-Hispanic white people, 56.8% were married, and the majority were overweight (32.7%) or obese (37.5%).

**Table 1:** Sample characteristics by sleep duration and sleep disturbance in the U.S. adult population, NHANES 2007–2018

Characteristics	All	Sleep duration^1^				Sleep disturbance		
		Short	Recommended	Long	*p*-value^2^	Yes	No	*p*-value^2^
Unweighted (n)	23,749	7483	13,341	2925		5956	17,793	
Total weighted %	100	31.5	56.1	12.3		25.1	74.9	
**Age, years %**					< 0.001			< 0.001
20–29	17.8	15.8	17.1	26.3		10.7	20.4	
30–39	18.7	21.1	17.9	17.0		16.4	19.6	
40–49	20.3	23.1	20.5	12.3		20.9	20.0	
≥50	43.3	40.1	44.5	44.5		52.1	40.0	
**Sex %**					< 0.001			< 0.001
Male	47.3	51.0	46.9	40.4		39.7	50.1	
Female	52.7	49.2	53.1	59.6		60.3	49.9	
**Race/Ethnicity %**					< 0.001			< 0.001
Hispanic	14.3	15.2	13.6	16.1		9.4	16.2	
Non-Hispanic White	66.5	59.1	70.3	63.7		74.2	63.6	
Non-Hispanic Black	11.0	17.1	8.2	11.1		9.8	11.4	
Other race	8.2	8.6	7.8	9.1		6.5	8.8	
**Educational Level %**					< 0.001			< 0.001
Less than 12th grade	14.2	16.6	12.4	17.8		12.2	14.9	
High school diploma	21.6	24.5	19.2	27.8		22.7	21.2	
Some college	31.3	33.4	30.2	32.3		35.1	29.9	
College graduate or above	32.9	25.6	38.2	22.0		30.0	34.0	
**Marital Status %**					< 0.001			< 0.001
Never married	18.0	18.4	16.8	23.7		16.4	18.7	
Married	56.8	53.7	60.5	44.7		52.9	58.3	
Divorced/widowed/separated	17.0	19.3	15.4	19.7		23.4	14.5	
Living with a partner	8.2	8.7	7.3	12.0		7.3	8.5	
**BMI, kg/m** ^**2**^ **%**					< 0.001			< 0.001
Underweight (< 18.5)	1.5	10.9	2.7	4.1		2.2	3.1	
Normal (18.5–24.9)	28.3	40.0	36.1	38.0		27.7	36.6	
Overweight (25.0-29.9	32.7	23.7	31.9	27.6		26.4	32.5	
Obese (≥ 30)	37.5	25.4	29.3	30.3		43.6	27.7	
**Physical Activity** ^**3**^ **%**					< 0.001			< 0.001
Active	65.9	66.8	66.8	58.8		62.3	67.3	
Not active	34.1	33.2	33.2	41.2		37.7	32.7	

*Abbreviations* %, weighted percentages; BMI, body mass index

^1^ Sleep duration: short (≤ 6 h), recommended (> 6 to < 9 h), long (≥ 9 h)

^2^ *P*-values are from chi-square test

^3^ Physical activity (active): participated in at least 150 min per week of moderate-intensity aerobic physical activity

Regarding sleep duration, 31.5%, 56.1%, and 12.3% reported sleeping for short (≤ 6 h), recommended (> 6 to < 9 h), and long (≥ 9 h) duration, respectively. The mean sleep duration was 7.1 ± 1.5 h. Participants with long sleep duration were more likely to be female, younger (20–29 years), Hispanic people, never married, and obese. Participants 50 years and older, female, non-Hispanic white people, divorced/widowed/ separated, and obese were more likely to report sleep disturbances compared to their counterparts. One in every four (25.1%) participants had told a health professional that they had trouble sleeping (sleep disturbance). Supplementary Table A.2. shows sample sizes for the number of participants whose CVD biomarkers were used in data analysis.

### Associations between sleep duration and biomarkers

Table 2 displays the associations between sleep problems and CVD biomarkers. In the crude model, we found increased odds for abnormal CRP (OR = 3.35, 95% CI = 1.26–8.90), HDL (OR = 1.33, 95% CI = 1.18–1.50), HbA1c (OR = 1.40, 95% CI = 1.18–1.67), blood glucose (OR = 1.18, 95% CI = 1.00-1.40), and insulin (OR = 1.32, 95% CI = 1.12–1.55) among those who reported short sleep duration compared to participants who reported the recommended sleep duration. Study participants who reported long duration had increased odds of having abnormal CRP (OR = 7.21, 95% CI = 2.39–21.73), HbA1c (OR = 1.60, 95% CI = 1.22–2.11), and blood glucose (OR = 1.46, 95% CI = 1.13–1.88), compared to participants in the recommended sleep duration category.

**Table 2:** Associations between sleep problems and abnormal CVD biomarkers in the U.S. adult population, NHANES 2007–2018

	Odds Ratio (95% Confidence Intervals)				
Abnormal biomarkers^1^	Sleep duration^2^			Sleep Disturbance	
(Total sample size)	Short	Recommended	Long	No	Yes
**Abnormal CRP (n = 6,371)**					
Crude model	3.35 (1.26–8.90) **	Ref	7.21 (2.39–21.73) **	Ref	1.49 (0.55–4.05)
Adjusted model ^3^	3.08 (1.18–8.05) **	Ref	6.12 (2.19–17.15) **	Ref	1.32 (0.45–3.85)
**Abnormal HDL (n = 23,445)**					
Crude model	1.33 (1.18–1.50) **	Ref	0.99 (0.81–1.20)	Ref	0.90 (0.80-1.00)
Adjusted model ^3^	1.20 (1.05–1.39) *	Ref	0.99 (0.81–1.20)	Ref	0.96 (0.83–1.12)
**Abnormal LDL (n = 10,995)**					
Crude model	1.01 (0.91–1.13)	Ref	0.87 (0.72–1.04)	Ref	1.09 (0.96–1.23)
Adjusted model ^3^	0.98 (0.87–1.10)	Ref	0.92 (0.76–1.12)	Ref	0.97 (0.84–1.11)
**Abnormal TG (n = 11,161)**					
Crude model	1.14 (0.98–1.31)	Ref	1.08 (0.92–1.26)	Ref	1.31 (1.14–1.50) **
Adjusted model ^3^	1.11 (0.95–1.29)	Ref	1.11 (0.94–1.30)	Ref	1.18 (1.01–1.37) *
**Abnormal HbA1c (n = 23,710)**					
Crude model	1.40 (1.18–1.67) **	Ref	1.60 (1.22–2.11) **	Ref	1.40 (1.16–1.70) **
Adjusted model ^3^	1.25 (1.05–1.49) **	Ref	1.54 (1.09–2.17) **	Ref	1.16 (0.95–1.42)
**Abnormal Glucose (n = 11,410)**					
Crude model	1.18 (1.00-1.40) **	Ref	1.46 (1.13–1.88) **	Ref	1.45 (1.22–1.72) **
Adjusted model ^3^	1.09 (0.91–1.29)	Ref	1.45 (1.07–1.95) *	Ref	1.24 (1.04–1.49) *
**Abnormal Insulin (n = 6,082)**					
Crude model	1.32 (1.12–1.55) **	Ref	1.26 (0.99–1.62)	Ref	1.32 (1.13–1.53) **
Adjusted model ^3^	1.24 (1.03–1.51) *	Ref	1.25 (0.89–1.77)	Ref	1.09 (0.91–1.31)

*Abbreviations* CVD: cardiovascular disease, Ref: Reference group, CRP: C-reactive proteins, Hb1Ac: glycohemoglobin,

HDL: high-density lipoproteins, LDL: low-density lipoproteins, TG: Triglycerides, Glucose: blood glucose level

^1^ Cutoff for cardiovascular biomarkers: CRP: C-reactive proteins (≥ 3 mg/dL), Hb1Ac: glycohemoglobin (≥ 6.5%), HDL: high-density

lipoproteins (≤ 50 mg/dL), LDL: low-density lipoproteins (≥ 130 mg/dL), Triglycerides: (≥ 150 mg/dL), Glucose:(≥ 126 mg/dL),

Insulin: (≥ 10.57uU/mL).

^2^ Sleep duration: short (≤ 6 h), recommended (> 6 to < 9 h), long (≥ 9 h)

^3^ Adjusted model included age, sex, educational level, race/ethnicity, marital status, BMI, and physical activity

* Denotes *P*-values ≤ 0.05; ** Denotes *P*-values ≤ 0.01

Bolded odds ratios and 95% confidence intervals indicate statistical significance

After adjusting for sociodemographic and lifestyle behavior, the odds of having an abnormal CRP level were higher among participants who reported both short (aOR = 3.08, 95% CI = 1.18–8.05) and long (aOR = 6.12, 95% CI = 2.19–17.15) sleep duration compared to participants who reported the recommended sleep duration. Participants who reported short sleep duration had higher odds of having abnormal levels of HDL (aOR = 1.20, 95% CI = 1.05–1.39), HbA1c (aOR = 1.25, 95% CI = 1.05–1.49), and insulin (aOR = 1.24, 95% CI = 1.03–1.51) compared to the recommended group. Long sleep duration was significantly associated with higher levels of HbA1c (aOR = 1.54, 95% CI = 1.09–2.17), and blood glucose (aOR = 1.45, 95% CI = 1.07–1.95) in the adjusted model. Participants with diabetes, hypertension, and high cholesterol were excluded from the adjusted models.

### Associations between sleep disturbance and biomarkers

Study participants who reported sleep disturbance had higher odds of abnormal triglycerides (OR = 1.31, 95% CI = 1.14–1.50), HbA1c (OR = 1.40, 95% CI = 1.16–1.70), blood glucose (OR = 1.45, 95% CI = 1.22–1.72), and insulin (OR = 1.32, 95% CI = 1.13–1.53) in the crude model (Table 2). After adjusting for sociodemographic and lifestyle behavior, participants who reported trouble sleeping had higher odds of abnormal triglyceride (aOR = 1.18, 95% CI = 1.01–1.37), and blood glucose (aOR = 1.24, 95% CI = 1.04–1.49) levels. Results from the tests for interactions showed an interaction between sleep duration and age, as well as sleep disturbance and age for CRP (*p* < 0.001).

### Sensitivity analysis

We calculated E-values for all statistically significant associations in the adjusted models (Table 2). A large E-value implies that substantial unmeasured confounding will be needed to explain away an observed association, and vice versa. The sensitivity analysis revealed that unmeasured confounding cannot easily explain away these observed associations. For instance, an E-value of 5.28 was obtained for the association between short sleep duration and CRP. This means that the observed association between short sleep duration and CRP could be explained away by an unmeasured confounder associated with both short sleep duration and CRP by a risk ratio of 5.28-fold each, above and beyond the measured confounders. An E-value of 12.16 was calculated for the association between long sleep duration and CRP, while the association between short sleep and HDL had an E-value of 1.37. The association between short sleep and insulin had an E-value of 1.46.

## Discussion

This study examined associations of sleep duration and sleep disturbance with CVD biomarkers in the U.S. adult population using data from the NHANES survey (2007 to 2018). One in four study participants reported sleep disturbance. Short sleep duration was positively associated with abnormal HDL, CRP, HbA1c, and insulin, while abnormal CRP, HbA1c, and blood glucose were positively associated with long sleep duration in the adjusted model. Triglycerides and blood glucose were positively associated with sleep disturbances in the adjusted model.

The percentage of study participants (25%) who reported having sleep disturbances in this study was like that reported in previous studies among adults in the U.S. (29.8%, 27.7%) [22, 23]. This finding underscores the importance of the problem among Americans. Further scientific inquiries will be pivotal in understanding the factors leading to sleep disturbance and interventions to overcome the problem.

We noted that short sleep duration increased the odds of abnormal HDL levels. This is in contrast with a recent systematic review on the association between sleep duration and abnormal lipid profile, where short sleep duration was not associated with HDL [24]. Nevertheless, other cross-sectional studies reported that short sleep duration was associated with abnormal HDL [25, 26]. Low HDL has been associated with an elevated risk of CVD, such as atherosclerotic CVD [27]. HDL’s cardiovascular defensive effect is attributed to its role in transporting excess cholesterol originating from the peripheral tissues to the hepatic system through reverse cholesterol transport [27]. In addition, low levels of HDL may have antioxidant, anti-inflammatory, and antithrombotic properties, which are likely to contribute to these athero-protective effects [28]. Although we cannot provide evidence of how long the participants had been exposed to short sleep duration due to the cross-sectional nature of the data used in this study, participants who reported short sleep duration may have experienced it for longer persistent periods exposing them to low HDL levels. However, recent studies have revealed contrasting findings [27, 29, 30]. Hence, more clinical trials are needed to investigate the causal pathways between HDL and CVD risk.

Similar to our findings on the association between blood glucose and triglycerides with sleep disturbances, other studies also reported that abnormal blood glucose [31] and triglycerides [31] were associated with sleep disturbances. Various sleep disorders are associated with high blood glucose levels and poor glycemic control [32, 33]. Insufficient sleep can cause increased glucose intolerance, thus increasing the risk of diabetes mellitus [34]. A systematic review on short sleep duration and the risk of developing insulin resistance reported similar findings and noted the limited understanding of the mechanisms involved [35]. The association between sleep duration and disturbance with glucose metabolism is not well understood since most related studies are observational [36]. However, these sleep disorders appear to shift morning cortisol levels and sympathovagal balance, thus increasing hepatic glucogenesis and reducing insulin sensitivity [36]. In addition, sleep disturbances can cause dysregulation of the sympathetic and parasympathetic regulation of pancreatic function [37]. Thus, strategies to ensure that sleep time is uninterrupted and adequate can improve the quality of sleep, create a balanced glucose metabolism, and curb long-term complications of insulin resistance, such as type 2 diabetes.

Our study findings revealed that a short sleep duration increased the odds of abnormal insulin levels in the adjusted model. This was consistent with earlier research studies, which show that short sleep duration is associated with insulin resistance [38, 39]. Insufficient sleep can result in increased glucose intolerance, thus increasing the risk of diabetes mellitus [38, 40]. It is also possible that elevated CRP levels have an underlying impact on the association between short sleep duration and prediabetes [41]. Sleep disorders appear to shift morning cortisol levels and sympathovagal balance, thus increasing hepatic glucogenesis and reducing insulin sensitivity [36]. Thus, strategies to ensure that sleep time is uninterrupted and adequate can be beneficial to ensure optimal metabolic health, create balanced glucose metabolism and curb long term complications of insulin resistance such as type 2 diabetes.

Our study revealed that both short and long sleep duration are positively associated with higher concentrations of CRP, a finding similar to those of studies in the U.S [6], Korea [42], and Taiwan [43]. Short sleep duration increases sympathetic nervous system activity, causing elevated CRP concentrations [44]. CRP is the primary marker of proteins that are responsible for the response to inflammatory stimuli; hence, it is useful in the prediction of myocardial infarction and stroke [6]. CRP also promotes the secretion of inflammatory mediators that increase the uptake of LDL by endothelial cells [44]. Proinflammatory cytokines high in obese and diabetic patients are suspected to be key players in sleep pathologies such as sleep fatigue and apnea. Thus, long sleep duration increases proinflammatory cytokines, and the cytokines increase CRP concentrations and promote the development of CVD [6].

### Strengths and limitations

The strength of this study was in the increased power based on the large sample size that was nationally representative and examined the associations between sleep measures and over six CVD biomarkers. The study also had some limitations. First, sleep duration and sleep disturbance in this study were self-reported and may, therefore, be inaccurate due to recall bias. The question used to assess sleep disturbance asked if the participant had ever told a health professional that they had trouble sleeping. This, therefore, depends on their recollection and may therefore be inaccurate. A recent study that compared a self-reported assessment of sleep problems and an objective assessment (actigraphy) reported a low level of agreement (57%) between the two methods [45]. The study evidenced the low level of accuracy in self-reported sleep problems. For sleep duration, participants were asked about their average sleep duration on weekdays/workdays. Data was not available for weekends/non-workdays sleep duration. Since sleep is usually longer and less disturbed on weekends/non-workdays [46], the findings of our study do not apply to weekends/non-workdays and must be interpreted in this context. Because of the unavailability of data, we could not account for the standard time clock changes in sleep duration and sleep disturbance. Second, the cross-sectional design has limits in making causal inferences in the relationship between sleep problems and CVDs. Third, the sample sizes for participants with some biomarkers, such as CRP, were small but significant, which could have exaggerated or attenuated the observed associations between sleep problems and biomarkers. Prospective evaluations using more sensitive scales on sleep problems will be useful for a clearer understanding of how they are associated with CVD risk. We recommend that future studies objectively measure sleep problems using validated methods, such as actigraphy.

### Study implications

The findings of this study are imperative in informing researchers to better understand the potential mechanisms between sleep problems and CVD biomarkers. The inflammation and HDL biomarkers may be potential mediators given the supporting evidence from the literature. These should be closely monitored for patients suffering from sleep disorders. Additionally, the findings of this study are of clinical importance in informing the assessment of CVD risk-enhancing factors to guide decision-making for comprehensive lifestyle interventions such as healthy diet, physical activity, and healthy sleeping habits among adults [47]. The Healthy People 2030 asserts the need for public awareness of the benefits of a healthy regular sleep schedule in a safe sleeping environment [48]. Proactive measures to ensure optimum sleep duration and minimize sleep disturbances by switching off electronic devices, avoiding stimulant drinks and foods at bedtime, consistent bed timing, and a serene and comfortable bedroom temperature are essential for healthy sleep. As part of medical management, periodic screening of CVD biomarkers will promote early detection of CVDs, particularly among individuals diagnosed with sleep disorders.

## Conclusions

Short and long sleep durations were positively associated with abnormal CRP, HDL, HbA1c, blood glucose, and insulin levels, whilst sleep disturbance was associated with abnormal triglyceride and blood glucose levels. Since sleep duration and sleep disturbances are modifiable measures, adopting healthy sleeping habits may create a balanced metabolism and reduce the risk of developing a CVD. Our study may provide insights into the relationship between sleep duration, sleep disturbance, and CVD risk.

### Electronic supplementary material

Below is the link to the electronic supplementary material.

Supplementary Material 1

Supplementary Material 2

## Data Availability

The datasets analyzed during the current study are available in the [NHANES] repository, [https://wwwn.cdc.gov/nchs/nhanes/Default.aspx]
