# Supplementary material for: Associations between sleep duration, sleep disturbance and cardiovascular disease biomarkers among adults in the United States
Source: BMC Public Health. 2024 Apr 2;24:947. doi: 10.1186/s12889-024-18381-5 (PMC10985959; doi:10.1186/s12889-024-18381-5)
Supplement: Supplementary file 1 — Supplementary Material 1 [file 12889_2024_18381_MOESM1_ESM.docx]

**Supplementary table A.2.** Sample sizes for the number of participants whose CVD biomarkers were used in data analysis.

| **Biomarkers** ^1^ | **Abnormal** | **Normal** |
| --- | --- | --- |
|  | **n (%)^2^** | **n (%)^2^** |
| **CRP (n=6,371)** | 72 (1.13) | 6,299 (98.87) |
| **HDL (n=23,445)** | 11,471 (48.93) | 11,974 (51.07) |
| **LDL (n=10,995)** | 3,415 (31.06) | 7,580 (68.94) |
| **TG (n=11,161)** | 2,578 (23.10) | 8,583 (76.90) |
| **HbA1c (n=23,710)** | 2,573 (10.85) | 21,137 (89.15) |
| **Glucose (n=11,410)** | 1,385 (12.14) | 10,025 (87.86) |
| **Insulin (n=6,082)** | 2,626 (43.18) | 3,456 (56.82) |

**Abbreviations:** CRP: C-reactive proteins, Hb1Ac: glycohemoglobin, HDL: high-density lipoproteins, LDL: low-density lipoproteins, TG: triglycerides.

^1^Cutoff for cardiovascular biomarkers: CRP: C-reactive proteins (≥3mg/dL), Hb1Ac: glycohemoglobin (≥6.5%), HDL: high-density lipoproteins (≤50mg/dL), LDL: low-density lipoproteins (≥130mg/dL), Triglycerides: (≥150mg/dL), Glucose:(≥126 mg/dL), Insulin: (≥10.57 uU/mL).

^2^Unweighted percentages
