# Supplementary material for: Associations between sleep duration, sleep disturbance and cardiovascular disease biomarkers among adults in the United States
Source: BMC Public Health. 2024 Apr 2;24:947. doi: 10.1186/s12889-024-18381-5 (PMC10985959; doi:10.1186/s12889-024-18381-5)
Supplement: Supplementary file 2 — Supplementary Material 2 [file 12889_2024_18381_MOESM2_ESM.docx]

| **Supplementary table A.1. Characteristics of included and excluded participants** | | |
| --- | --- | --- |
| **Characteristics** | **Included [n (%)] (n=23,749)** | **Excluded [n (%)] (n=36,093) *** |
| **Age, years** |  |  |
| 20-29 | 3,737 (15.7) | 1,954 (17.7) |
| 30-39 | 4,214 (17.7) | 1,639 (14.9) |
| 40-49 | 4,342 (18.3) | 1,403 (12.7) |
| ≥50 | 11,456 (48.2) | 6,025 (54.7) |
| **Sex** |  |  |
| Male | 11,174 (47.0) | 18,455 (51.1) |
| Female | 12,575 (53.0) | 17,638 (48.9) |
| **Race/Ethnicity** |  |  |
| Hispanic | 5,966 (25.1) | 11,446 (31.7) |
| Non-Hispanic White | 9,522 (40.1) | 11,876 (32.9) |
| Non-Hispanic Black | 4,993 (21.0) | 8,369 (23.2) |
| Other race | 3,268 (13.8) | 4,402 (12.2) |
| **Educational Level** |  |  |
| Less than 12th grade | 5,174 (21.8) | 3,522 (32.1) |
| High school diploma | 5,201 (21.9) | 2,720 (24.8) |
| Some college | 7,228 (30.4) | 2,916 (26.6) |
| College graduate or above | 6,146 (25.9) | 1,811 (16.5) |
| **Marital Status** |  |  |
| Never married | 4,355 (18.3) | 2,090 (19.0) |
| Married | 12,579 (53.0) | 4,994 (45.4) |
| Divorced/widowed/separated | 4,923 (20.7) | 2,991 (27.2) |
| Living with a partner | 1,892 (8.0) | 919 (8.4) |
| **BMI, kg/m^2^** |  |  |
| Underweight (<18.5) | 358 (1.5) | 9,988 (34.51) |
| Normal (18.5-24.9) | 6,465 (27.2) | 9,064 (31.3) |
| Overweight (25.0-29.9 | 7,824 (32.9) | 4,968 (17.2) |
| Obese (≥30) | 9,102 (38.3) | 4,922 (17.0) |
| **Physical Activity** |  |  |
| Not active | 9,211 (38.8) | 6,680 (38.0) |
| Active | 14,538 (61.2) | 10,916 (62.0) |
| **Sleep duration** |  |  |
| Short | 7,483 (31.5) | 25,879 (71.7) |
| Recommended | 13,341 (56.2) | 7,879 (21.8) |
| Long | 2,925 (12.3) | 2,335 (6.5) |
| **Sleep disturbance** |  |  |
| Yes | 5,956 (25.1) | 3,295 (22.3) |
| No | 17,793 (74.9) | 11,495 (77.7) |

*Numbers (n) in the excluded column may not add up to 36,093 due to missing values
